# Myoelectric control of robotic lower limb prostheses: a review of electromyography interfaces, control paradigms, challenges and future directions

**DOI:** 10.1088/1741-2552/ac1176

**Published:** 2021-07-27

**Authors:** Aaron Fleming, Nicole Stafford, Stephanie Huang, Xiaogang Hu, Daniel P Ferris, He (Helen) Huang

**Affiliations:** 1 Joint Department of Biomedical Engineering, North Carolina State University, Raleigh, NC 27695, United States of America; 2 Joint Department of Biomedical Engineering, University of North Carolina at Chapel Hill, Chapel Hill, NC 27599, United States of America; 3 Department of Mechanical and Aerospace Engineering, University of Florida, Gainesville, FL 32611, United States of America; 4 J. Crayton Pruitt Family Department of Biomedical Engineering, University of Florida, Gainesville, FL 32611, United States of America; 5 Equal contribution as the first author.

**Keywords:** robotic lower limb protheses, neural–machine interface, EMG, gait and balance, human motor control

## Abstract

**Objective.:**

Advanced robotic lower limb prostheses are mainly controlled autonomously. Although the existing control can assist cyclic movements during locomotion of amputee users, the function of these modern devices is still limited due to the lack of neuromuscular control (i.e. control based on human efferent neural signals from the central nervous system to peripheral muscles for movement production). Neuromuscular control signals can be recorded from muscles, called electromyographic (EMG) or myoelectric signals. In fact, using EMG signals for robotic lower limb prostheses control has been an emerging research topic in the field for the past decade to address novel prosthesis functionality and adaptability to different environments and task contexts. The objective of this paper is to review robotic lower limb Prosthesis control via EMG signals recorded from residual muscles in individuals with lower limb amputations.

**Approach.:**

We performed a literature review on surgical techniques for enhanced EMG interfaces, EMG sensors, decoding algorithms, and control paradigms for robotic lower limb prostheses.

**Main results.:**

This review highlights the promise of EMG control for enabling new functionalities in robotic lower limb prostheses, as well as the existing challenges, knowledge gaps, and opportunities on this research topic from human motor control and clinical practice perspectives.

**Significance.:**

This review may guide the future collaborations among researchers in neuromechanics, neural engineering, assistive technologies, and amputee clinics in order to build and translate true bionic lower limbs to individuals with lower limb amputations for improved motor function.

## 1. Introduction

A human controlling a prosthetic limb as if it were their own biological limb has fascinated biomedical researchers for many decades [1–6]. At the center of this idea is a direct link between the human nervous system and the prosthesis actuators, allowing for commands from the user to the prosthetic limb. Electromyography (EMG) provides an additional way to decode peripheral efferent signals from muscles in the residual limb [7]. EMG signals are common control signals for powered upper limb prostheses and have been in use for over 50 years [8]. In contrast, EMG control of robotic lower limb prostheses is still in its infancy. This is largely because: (a) motorized, robotic lower limb prostheses have only been practical as devices in the past decade, and (b) autonomous control of robotic lower limb prostheses has been sufficient to support basic locomotive activities in amputee users [9–15].

The time is ripe to develop myoelectric control of lower limb prostheses to maximally restore motor function of individuals with lower limb amputations. The mechatronics of robotic lower limb prostheses have become more mature, practical, and accessible [15–17], yet these modern, robotic devices are still limited in function, partly because the devices are preprogramed autonomous machines [10, 11, 18–22] unable to directly take user input. Current autonomous prosthesis controllers are sufficient to actively assist cyclic stepping motions in predictable environments (e.g. a clean floor in clinics), however they are inadequate to actively assist versatile daily tasks that require coordination with user intent (e.g. anticipatory postural adjustments in standing or walking, performing leisure activities) [23, 24]. They also do not provide adaptation to varying, unconstructed environments and task contexts (e.g. change of load carriage or walking on uneven terrains) [4, 25]. While increasingly complex autonomous control designs are being developed to incrementally address these draw-backs, myoelectric control, on the other hand, can be a simple and viable solution to resolve these limitations because the human motor control system is highly flexible and adaptable to changing tasks and environments.

Publications on EMG control of robotic lower-limb prostheses have started to emerge and accumulate in the last decade [4, 5, 26–29]. Decoding algorithms and control frameworks have significantly advanced since the early foundational EMG control analysis [1, 30, 31]. These pioneering studies explored different EMG decoding algorithms and control frameworks, brought forth novel functionality in robotic prosthetic legs which cannot be easily achieved by autonomous control, and showed feasibility and promise in amputee testing. However, none of these existing methods have been adopted by commercial robotic prostheses so far. As myoelectric control of robotic lower-limb prostheses is a growing topic of interest in the field, and review of the related literature has been very limited, there is a pressing need to summarize existing methods on this topic, understand challenges facing translation to the community, and highlight their potential applications and future directions.

Hence, this paper aims to summarize the literature related to EMG control of robotic lower-limb prostheses and highlight existing challenges, potential solutions, and opportunities related to its widespread clinical implementation. One goal of this review is to emphasize the need for more fundamental research on the neuromechanics of lower-limb amputees using neurally controlled prostheses. A second goal is to highlight the need for innovations in neural–machine interfacing technologies in lower-limb prostheses. Lastly, we hope to inspire more collaborations across disciplines to further our understanding on the potential and limitations of EMG control of robotic lower-limb prostheses, compared to current autonomous control. To address our goals, we first review the different surgical approaches/muscle nerve configurations and how they could influence EMG control. We then summarize the current methods for measuring EMG and the existing EMG control paradigms. Finally, we address current opportunities for EMG control to improve autonomous Prosthesis control. The resulting knowledge may provide a novel control framework for robotic lower-limb prostheses, shared by both autonomy and humans, to maximize the mobility of individuals with lower-limb amputations in the future.

## 2. Literature review

Considerations of the biological configuration of residual muscles, existing sensor technology and current control strategies will be needed to advance the field of EMG prosthesis control. This section reviews the current state of each of those areas to provide a full perspective of the state of EMG control in lower-limb prosthetics. We start with residual muscle configurations to summarize existing amputation procedures and how they could impact EMG residual muscle signal quality. Section 2.2 reviews current methods for measuring residual muscle EMG inside the prosthetic socket. Section 2.3 summarizes current EMG control paradigms, in which we focus on supervisory control (i.e. hierarchical combination of an EMG decoder for locomotion mode recognition with state-machine-based autonomous control) and direct control (i.e. continuous EMG control of prosthetic joint mechanics). Within each control paradigm, we layout considerations/approaches as well as evaluation methods and reported results. Tables 1 and 2 in the appendix provide additional information about study methods and controller information for the reviewed studies.

### 2.1. Amputated muscle/nerve configuration

The configuration of the muscle–nerve attachment (i.e. to bone or tendon) in the residual limb determines how existing biological signaling pathways can be used for prosthetic feedback and neuromuscular control [32–34]. Many factors, such as the cause of amputation (e.g. traumatic or dysvascular), residual limb length and shape, and subsequent muscle atrophy, can influence existing number of motor units, proprioceptors, and afferent neurons, which alter muscle fiber function and quality [35–38]. The type of surgical technique used for limb amputation is crucial for preservation of muscle tone and length, motor unit recruitment, and proprioception. For example, residual muscles must be stabilized to either muscle or bone at appropriate tensions [39, 40] because insufficient or too high tension can lead to atrophy, contractures, and/or pain. These resulting issues can affect residual muscle activity [39–41] and the quality of EMG signals in prosthesis control.

Traditional surgical techniques for lower limb amputations have had little evolution and do not consider the neural interface for prosthesis control [42]. The most common surgery for transtibial and transfemoral amputees discards distal tissue around the amputation site and fixes isolated muscle bellies through a combination of myodesis and myoplasty [39, 43, 44]. Myoplasty sews opposing muscle groups together while myodesis attaches muscles directly to bone [39]. The nerves are transected and positioned in soft tissue away from scar tissue, the incision, or areas subject to prosthetic socket irritation, with the goal of minimizing painful neuromas [39]. Residual muscles after traditional amputation surgery can still be activated by the brain and spinal cord, but the EMG patterns during walking are often different from the patterns in physically intact humans [45–47].

Recently, novel surgical techniques have been developed that consider the human–machine interface of a powered lower limb prosthesis. The goal of the surgical techniques is not just to reshape the residual limb, but to improve the neural interface for adaptable, reliable neuromuscular control of lower limb prostheses in dynamic real-world environments.

One surgical technique, the agonist–antagonist myoneural interface (AMI), attempts to use the body’s natural mechanisms for proprioceptive feedback to enhance prosthetic control and embodiment. In the AMI, surgeons reconnect agonist–antagonist residual muscle pairs to restore reciprocal muscle function [48]. When the agonist contracts, the mechanical linkage stretches the antagonist and vice versa. Such reciprocal contractions engage length and force receptors in both muscle-tendon units, resulting in a more natural sensation of position and velocity for improved motor control of residual muscles [32, 48, 49]. One individual who received the AMI procedure produced more isolated contractions of antagonist residual muscles and improved stability in gait-related tasks when using an EMG controlled two-degree-of-freedom prosthetic ankle compared to amputees without the AMI [32]. The individual also exhibited reflexive prosthesis motions indicating a higher level of embodiment. In order to produce this antagonist mechanical linkage for individuals who have already received a ‘traditional’ amputation surgery, a regenerative neural interface has been proposed to implement AMI through the use of targeted muscle reinnervation (TMR) and muscle grafts [50, 51].

TMR is a surgical technique that aims to restore neuromuscular control sources in amputees by transferring residual nerves to muscles that are no longer biomechanically functional [33, 34, 52]. The reinnervated muscles act as a biological amplifier to restore EMG recording sites for the missing joint control [33, 34]. For example, the tibial nerve branch for transfemoral amputees can be connected to the semitendinosis and the common peroneal nerve branch can be inserted into the long head of the biceps femoris [53]. The EMG signals from these reinnervated thigh muscles can convey neuromuscular control signals for the prosthetic ankle joint. Combining peripheral nerve surgery with EMG based control strategies for prosthetics has led to more coordinated control of multi-jointed prosthetic devices. The majority of studies using TMR have been on individuals with upper-limb amputations [33, 54, 55], but there is increasing focus on shifting to individuals with lower-limb amputations [53].

### 2.2. Neural interfaces

One of the most critical aspects of neural prosthesis control is the accurate and robust sensing of neuromuscular activity (i.e. the control input). The limb–socket interface of lower-limb prostheses, which are subject to weight bearing forces, sweat accumulation, changes in limb volume, make EMG sensing challenging (discussed more in section 3). A majority of existing studies on myoelectric control of lower limb prostheses have used bipolar surface EMG electrodes to record neuromuscular control signals. One major challenge with this approach is the attachment of EMG electrodes within the prosthetic socket or liner for reliable EMG recordings without compromising socket suspension or user comfort. One study developed several socket-EMG interfaces that integrated commercial EMG electrodes on the prosthesis socket directly [56]. Additionally, fluctuations in residual limb volume over time can compromise reliable skin contact with the sensor inside the socket. Recent studies have used low profile, neonatal EMG sensors within the prosthetic socket for successful myoelectric prosthesis ankle control [57, 58]. A novel prosthetic liner with embedded dome electrodes and conductive textile fibers [59] can ease the sensor placement and wire management and yield reliable skin-electrode contact. Furthermore, new flexible, low-profile EMG sensors [60] have the potential to be fabricated directly within the prosthetic liner, ensuring comfort and reducing skin contact problems that arise from limb volume fluctuations.

Another challenge with myoelectric control of lower limb prostheses can be the placement of bipolar EMG electrodes to target specific residual muscles. Although the volume of lower-limb muscles is relatively large compared to those in the upper limbs, identifying specific muscles is often challenged by atrophy of residual musculature and lack of knowledge on amputation procedure. Fite *et al* used principal component analysis of surface EMG to reduce the effect of variation in measured residual muscle activity caused by differences in sensor placement across days [27]. This provided some success at standardizing the myoelectric signals for prosthetic control.

Implantable EMG sensor interfaces can mitigate limitations accompanying bipolar surface electrodes. Wireless intramuscular EMG sensors have been developed recently to transmit muscle activity signals from residual muscles to the prosthesis without any transcutaneous leads [61–66]. This interface has significant potential to target specific residual muscles not reachable with surface EMG and could be surgically implanted in parallel with other surgical procedures such as osseointegration or nerve reinnervation. These invasive neural interfaces have been, however, primarily tested in upper limb amputees to date. We are aware of only one study that implanted wireless intramuscular EMG sensors in lower-limb amputees for prosthesis control [64]. Another promising technology is high-density, flexible surface EMG. It may provide more information and greater resolution of residual muscle activations for prosthesis control. High-density EMG was first used with amputees to confirm reinnervation of residual muscles [67, 68]. Other studies have used high-density EMG to remove motion artifacts [69, 70] in walking and measure muscular activity from ankle flexors/extensors and invertors/evertors for prosthetic control [71]. High-density EMG shows significant potential for future prosthesis control development through integration of individual motor unit activations with prosthetic control, targeting specific muscle locations easily, and removing artifacts caused by movement.

### 2.3. EMG control paradigms

**Figure 1.**
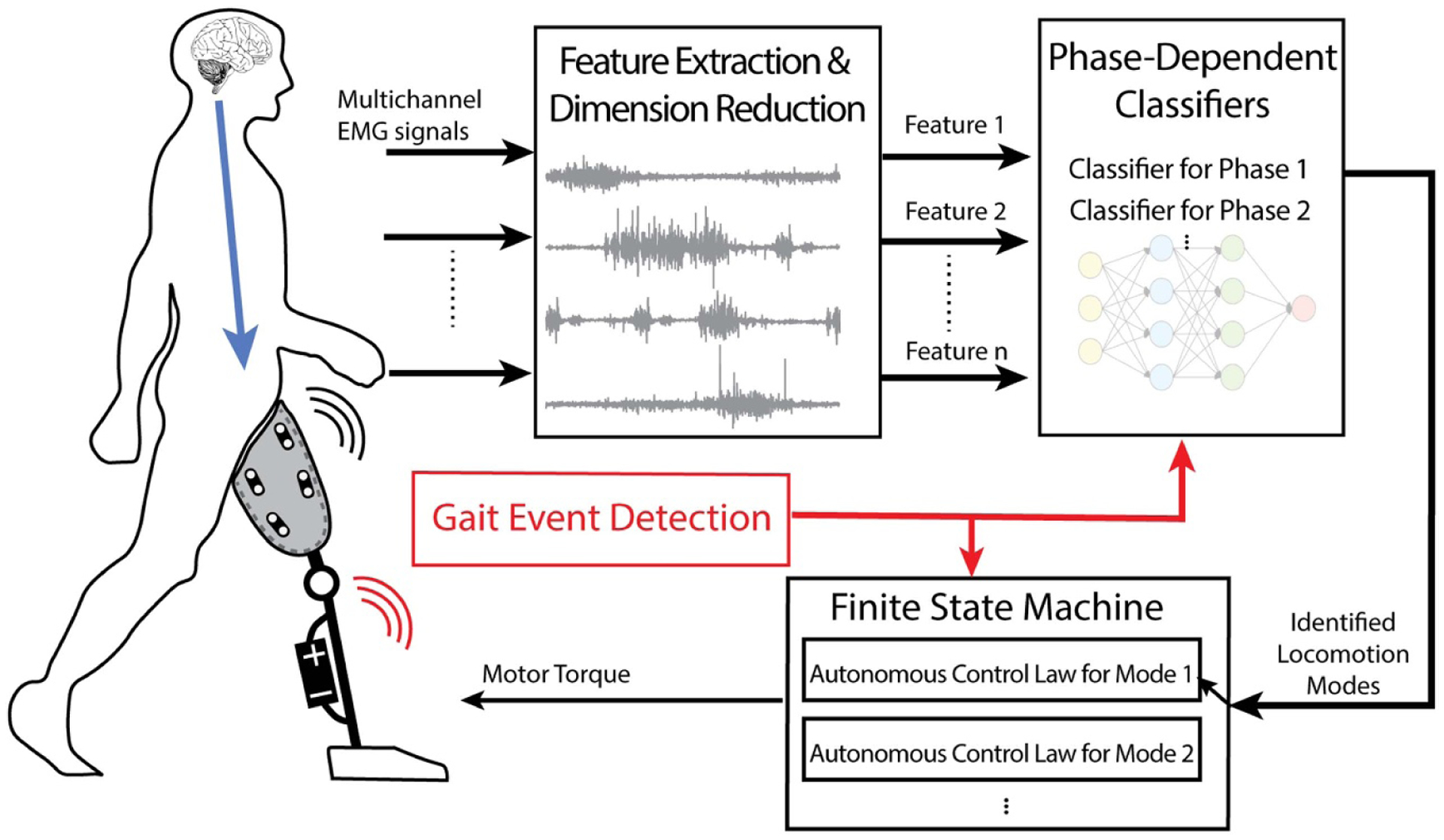
Supervisory EMG control paradigm for robotic lower-limb prosthesis. In supervisory EMG control, EMG signals and gait events are used to classify the user’s locomotion mode (such as level-ground walking, stair ascent/descent, ramp ascent/descent). The classifier’s decision determines transitions between the predefined finite-states and thus the specified low-level control (e.g. impedance control) for prosthesis operation associated with the identified locomotion mode.

**Figure 2.**
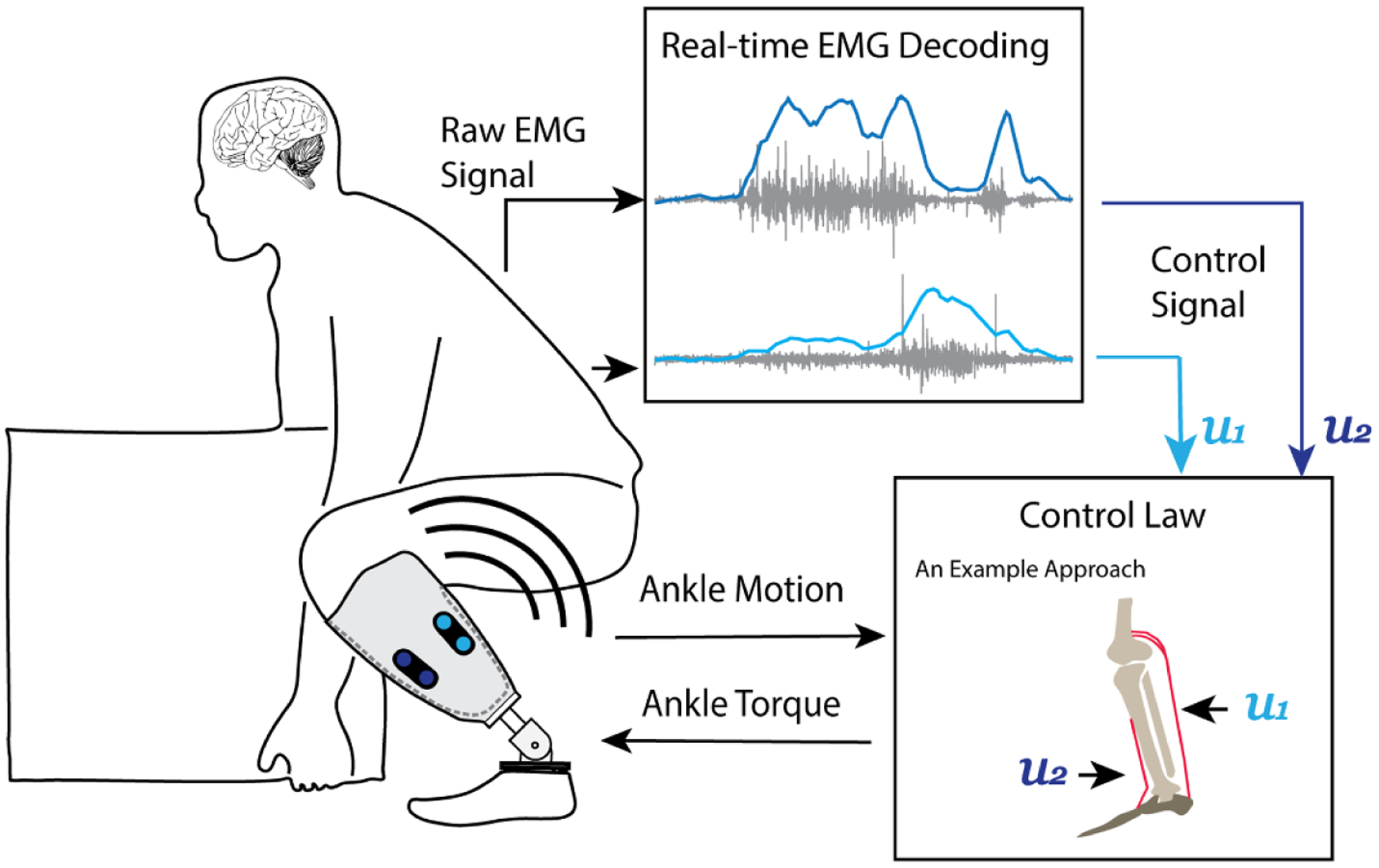
Direct EMG control paradigm for robotic lower-limb prosthesis. In direct EMG control, the magnitude of of EMG signals recorded from antagonistic residual muscles directly and continuously modulate the prosthesis joint dynamics. Various control laws can be used to continuously map EMG activity to ankle control torque to drive prosthesis dynamics. For example, EMG magnitude of residual ankle antagonistic muscles (u1 and u2) can activate an EMG-driven musculoskeletal model to estimate intended ankle control torque.

Current commercialized robotic prosthetic legs (e.g. PowerKnee^™^, Össur, Iceland; EmPower, Otto bock, Germany) do not rely on active neuromuscular human input for control but instead use onboard kinetic/kinematic sensing to drive autonomous controllers for pre-programmed activities [72]. These commercial devices employ finite-state machines to adjust knee and/or ankle joint impedance or position the joints based on predefined states such as the gait phase (e.g. swing and stance) and locomotion mode (e.g. stair ascent and level-ground walking) [10, 11, 18]. Transitions between gait phases can be triggered by measurements of intrinsic sensors (e.g. a load cell or motion sensor) in the prosthesis, while transitions between locomotion modes often requires input from the human user (e.g. specific body motions measured by sensors) [14, 73–75]. Existing autonomous control approaches are sufficient to assist amputees walking in well-defined environments, but they are inadequate for unconstrained tasks that require dynamic user intent and/or adaptation with varying environments (e.g. trail hiking, jumping, catching objects). These limitations have sparked interest in the research community to develop neural/EMG control that might improve adaptability and versatility of robotic lower-limb prostheses. There have been two prominent approaches to integrate amputee users’ efferent neural signals (i.e. EMG signals) for lower-limb prosthesis control in the current literature: supervisory EMG control and direct EMG control (figures 1 and 2).

#### 2.3.1. Supervisory EMG control

In current commercial robotic lower limb prostheses, transitions between locomotion modes are achieved manually, which is cumbersome. Instead, supervisory EMG control automatically recognizes the user’s locomotion mode by EMG pattern recognition (figure 1). By monitoring EMG patterns, prostheses can hierarchically adjust low-level autonomous control (e.g. finite state machine) to switch control based on recognized locomotion modes [2, 4, 29, 76, 77]. Essentially, supervisory EMG controllers are built upon autonomous locomotion-mode-dependent prosthesis control, where the joint mechanics in each mode are dominated by the low-level autonomous control. In this manner, the supervisory EMG controller acts as a part of a finite-state machine and it adjusts the mechanics of prosthesis joints only at the locomotion mode transitions.

##### 2.3.1.1. Input signals

In past studies, researchers used EMG signals recorded from residual limb muscles as neural inputs for locomotion mode recognition [4, 29, 78–81]. EMG electrodes are typically placed on the residual limb based on intact muscle anatomical location, palpation, and visual inspection of EMG signals [11, 29, 82]. Because EMG pattern recognition classified the locomotion mode based on multi-channel signal pattern, cross-talk in EMG recordings did not significantly influence classification performance. Within the existing studies, as many as nine EMG electrodes on a residual thigh [4] or four electrodes on a residual shank [80] were used. Groups also experimented with augmenting classifier inputs with muscles above the amputation level (e.g. the gluteus maximus on transfemoral amputees or the thigh muscles of transtibial amputees) [4, 83, 84]. However, adding sensors to intact muscles requires additional sensors outside the prosthetic socket, increasing the complexity for daily use and sensor setup. Another group found TMR surgery on a transfemoral amputee enhanced myoelectric control information recorded from reinnervated residual muscles, improving prosthesis control [29, 85]. The participants with TMR surgery had around a 40% error reduction rate during virtual movements compared to the amputee participants without TMR [29]. Furthermore, pattern recognition that combined EMG signals with intrinsic mechanical measures (neuromuscular–mechanical fusion) further improved the accuracy and reliability of locomotion mode recognition [26, 73, 85]. This fusion-based approach outperformed the algorithm that solely used EMG or only used mechanical measurements as system inputs [26]. With this, a source selection study showed EMG signals were essential for accurate prediction of user locomotion mode transitions compared to mechanical measurements alone [78].

##### 2.3.1.2. EMG feature extraction and phase-dependent EMG pattern classification

EMG pattern recognition has been widely used for upper limb prosthesis control [3, 8, 86–89], but adjustments are necessary for lower-limb prosthesis control. For control of upper-limb prosthesis movements (e.g. hand open), the human user must attempt the hand motion and hold the posture of the phantom hand. During this period, the EMG signals are considered to be stationary, i.e. the distribution of the stochastic signals does not change, and therefore the EMG activation pattern is consistent for the classifier to identify the user intended motion for continuous prosthesis control. In contrast, EMG signals in the lower limbs during walking are non-stationary over a full gait cycle. As a result, a different EMG pattern recognition strategy is necessary for lower limb prosthesis control compared to upper limb prosthesis control. If we assume that: (a) gait EMG is quasi-stationary within a gait phase, and (b) EMG patterns recorded from residual muscles are different between locomotion modes, but consistent within the same mode, then a phase-dependent EMG pattern recognition strategy can be implemented. This approach has been enacted with a system consisting of multiple pattern classifiers, each corresponding to a gait phase [4] (figure 1).

In each phase, the pattern recognition includes feature extraction, dimension reduction (optional), pattern classification, and post processing of classification decisions (optional). Feature extraction is an important step for accurate pattern classification. Selected features from the input data sources should maximally extract information that can distinguish between locomotion modes (classes). Focusing on EMG features, time domain (e.g. number of zero crossings, mean absolute value, and slope sign change) [26, 29, 80, 90] and frequency domain (e.g. medium frequency [76], bi-spectrum [77]) features have been used previously. Additionally, adding autoregression coefficients for EMG features can account for potential signal degradation, fatigue, and motion artifacts [29, 73, 91–93]. Groups have also used dimension reduction techniques, such as principal component analysis, to reduce the dimension of feature vectors and prevent model overfitting [79, 83, 92, 94]. Other feature/source reduction methods explored in lower-limb prosthesis control include sequential forward and backward selection and minimum-redundancy-maximum-relevance algorithms [78]. The extracted features were fed to a pattern classifier for locomotion mode recognition. A variety of commonly used classifiers have been used, e.g. artificial neural networks, support vector machine (SVM), linear discriminant analysis (LDA), quadratic discriminant analysis (QDA), and dynamic Bayesian networks (DBNs). DBN can be combined with LDA to provide time history and feed forward information to the classifier [92]. The DBN model predicts the future locomotion model, while LDA labels the previous stride. This structure is especially helpful with EMG inputs because the classifier can re-learn EMG patterns over time or across multiple training sessions. Finally, a post-processing method, such as majority vote [26], has been considered to further reduce classification errors, but increased the system delay for real-time applications.

##### 2.3.1.3. EMG pattern recognition based prosthesis control

In practice, EMG or data fusion-based decoders require model training before applying them to real-time prosthesis control. Model training requires collection of labeled training data (i.e. input data with class labels), followed by establishing the parameters in the classifiers. Collecting enough training data for multiple conditions, such as sit-to-stand, ramps, stairs, and level-ground walking across multiple speeds could take hours, on top of time needed for tuning/customizing prosthesis control parameters for each user [29]. In addition, daily recollection of EMG training data for each individual user is required for reliable performance [29, 78]. A means for efficient and automatic training data collection could minimize users’ time and effort to calibrate the system [95].

During real-time operation, the trained EMG classifier estimates the locomotion mode, which triggers task state transitions in the finite-state machine for robotic leg control (figure 1). One challenge is EMG pattern recognition approaches are sensitive to EMG signal variability caused by disturbances (like motion artifacts and electrode location shifts over time/multiple sessions) or physiological changes (such as muscle fatigue) [80, 93, 96–99], which threatens the reliability of the supervisory EMG control system and user safety. Beyond re-training the pattern classifier, other solutions have been proposed to improve the robustness of locomotion mode recognition system. For example, classifiers with redundant EMG sensors can detect abnormal signals, reject corrupted EMG channels, and only select viable EMG signals and mechanical sensor inputs for robust performance [96]. Adaptive pattern recognition, which can update the parameters in the classifier while using it in real-time, can be another promising solution to allow for robust classifiers better equipped to handle real-world settings [92, 100]. Another challenge for supervisory EMG control is the definition of timing to trigger the switch for low-level prosthesis control mode [101]. Although EMG pattern recognition provides real-time decisions regarding the user’s locomotion mode, the low-level controller parameter only updates at one critical timing that is defined for each type of task transition. For example, Huang *et al* defined the critical timing for transitions from level-ground walking to stair ascent at the prosthesis foot toe-off before stepping on the staircase to ensure a smooth and safe switch of walking terrain in amputee users [26]. Table 1 in the appendix summarizes existing literature related to EMG-based locomotion mode recognition and supervisory control with detailed approaches used in each study. Note we only included the studies that tested the system on individuals with lower-limb amputations in this table.

##### 2.3.1.4. Performance/evaluation metrics

The performance of supervisory EMG control systems is typically evaluated by classification error/accuracy rate, the confusion matrix during steady state activity, and/or prediction accuracy of task transition and prediction time. The reported accuracy rate ranges from 75% to 99%. Usually task transition can be predicted accurately before the defined critical timings [26, 77, 92, 93]. The most common classification error is between ramps and level-ground walking [29, 92, 102]. However, how these engineering performance metrics influence the amputee user’s locomotion performance is unclear. Zhang *et al* systematically studied the influence of errors and delays in supervisory EMG control of robotic knee prostheses on human walking stability [101, 103]. The research found that not all errors disturb measured dynamic stability and the user’s perceived walking stability; it depends on the timing and cumulated mechanical work change around the prosthesis knee joint. The group also suggested a range of timing for switching prosthesis control mode that ensures user safety during terrain transitions [101].

Supervisory EMG controllers are inherently autonomous finite-state-machine-based controllers where the low-level autonomous control law dominates the joint mechanics. Even though the EMG signals are included in the control algorithm, the EMG control only functions during locomotion mode (i.e. state) transitions and the approach is inadequate to enable the prosthesis to assist tasks that have not been preprogramed in the low-level control. This approach is also problematic for tasks that do not readily conform to the autonomous finite-state-based controller (e.g. dancing, sports activities).

#### 2.3.2. Direct EMG control

While most lower-limb prosthesis controllers measure prosthesis activity or human muscle activity to inform a state prediction for autonomous control, direct EMG control uses active and continuous input from the human user muscle activity to determine prosthesis dynamics. Thus, direct EMG control mimics the biological neural control pathway in an intact musculoskeletal system. The efferent neural signals (EMG) of the residual agonist–antagonist muscle pairs are used to directly modulate prosthesis joint mechanics (i.e. impedance, angle, and/or torque) (figure 2). The prosthesis joint mechanics can be determined by the human feedforward neural output. This method has shown increasing success in improving various activity performance and postural control in a recent study [58]. Note that direct EMG control here is defined as a myoeletric control method for powered prostheses, which follows antagonistic muscle function around a joint for movement control. Therefore, non-biomimicry mappings of EMG signals to joint mechanics, such as neural networks, are not discussed in this section.

##### 2.3.2.1. EMG decoding methods and control

The inputs of the decoder for direct EMG control are the EMG signals recorded from residual antagonistic muscles. Most commonly, the magnitude of the EMG signals proportionally increases a prosthetic joint parameter [5, 104]. One challenge of this approach is that the antagonist residual muscle sometimes inadvertently contracts as the amputee intends to activate the agonist muscle only, causing a certain level of involuntary co-activation [27, 45]. Involuntary co-activation limits the ability of amputees to access portions of the control input space like isolated joint flexion or extension. To avoid this problem, one approach incorporates an EMG classifier in the direct EMG control scheme to identify the isolated intended joint motion (e.g. flexion vs extension) first [94]. This approach has been evaluated on individuals with amputations in the sitting position, but not during walking. Another solution first transforms the multiple-channel EMG inputs via principal component analysis [27] or non-negative matrix factorization [71] to obtain the isolated ‘motor primitive’ representing the voluntary control for each studied motion. These decoding algorithms may help amputees with involuntary co-activation [45]. However, it remains to be seen whether amputees are capable of generating more isolated residual muscle contractions given sufficient training.

A large portion of work has used impedance control laws, where neuromuscular activity modulate one or multiple joint impedance parameters (i.e. set stiffness, equilibrium position, etc). Initial efforts with impedance control used residual muscle activity to proportionally modulate equilibrium velocity (i.e. rate of change of the equilibrium point) [27, 94]. Subsequent studies incorporated relative co-activity from residual muscles to additionally modulate the set stiffness value in the impedance control law [27, 28, 48, 71]. These studies have shown the ability for amputees to volitionally modulate both stiffness and position of the prosthesis, showing promise for its commercial use. Another common output of the decoder is joint torque. While using the impedance control law described above, Fite *et al* also incorporated additional torque gain terms for the residual thigh flexors/extensors to proportionally generate control torque for a prosthetic knee [104]. This allowed the direct torque terms to be weighted depending on phase of gait (stance vs. swing) and for the impedance control to be more responsible for limb kinematics during the swing. Using a pneumatically actuated prosthetic ankle, Huang *et al* used EMG magnitude of the residual gastrocnemius (GAS) to proportionally modulate plantar-flexor torque [5, 105], and this control method has been extended to two agonist–antagonistic residual muscles to control both dorsi- and plantar-flexor torques [57, 58].

Musculoskeletal models are another possible means of EMG decoding method for direct EMG control (figure 2). The EMG magnitude, extracted from EMG signals of residual agonist–antagonist muscles, activates a virtual musculoskeletal model (similar to a human biological joint) to estimate the missing joint mechanics. However, this type of control has only been tested with a virtual ankle joint for transtibial amputees in a sitting position [81] and with able-bodied individuals walking with a bent-knee adapter [106].

##### 2.3.2.2. Activity evaluation

The gold-standard task to evaluate direct EMG control paradigms in the literature has been locomotion. For transfemoral amputees, direct EMG control has been tested for over-ground walking [27, 107] and stair ascent [104]. These preliminary studies with an individual amputee showed potential for amputees to adapt residual thigh muscles to control prosthetic knee torque during cyclic movements like walking. For transtibial amputees, direct EMG control has been tested in over-ground walking using the residual GAS [5, 105]. Amputees successfully adapted their residual muscle activation once given feedback of the prosthetic ankle state. Clites *et al* incorporated multiple residual muscles into prosthesis control for stair ascent and descent for transtibial amputees [48]. Direct EMG control was also tested in stair ascent/descent and over-ground walking on a transtibial amputee after receiving an AMI surgery [48]. The AMI recipient demonstrated restored reflexive muscle activity and improved prosthesis embodiment, compared to amputees without AMI procedures using direct EMG control.

One of the benefits for direct EMG control is that it is not constrained to rhythmic locomotor tasks. Instead, direct EMG enables prosthesis assistance for a variety of activities in daily living. Unfortunately, there has been limited work to understand amputees’ ability to use direct EMG control for other daily activities. Rogers *et al* demonstrated the ability for EMG control of a novel powered ankle prosthesis to augment rock climbing in a person with transtibial amputation [108]. One preliminary study investigated direct EMG control use during situations with expected perturbations [57]. This study showed a transtibial amputee could produce anticipatory postural adjustments on an EMG-controlled prosthetic ankle to significantly improve stability after a perturbation. They also studied the ability for a transtibial amputee to control a variety of standing postural control tasks like quiet standing on firm and compliant surfaces as well as load transfer tasks [58]. The results demonstrated the ability for an amputee to significantly improve bilateral EMG activation synchronization and standing postural control with direct EMG control of a prosthesis ankle after extended, guided training with a physical therapist. These aforementioned activities have never been demonstrated by autonomous or EMG supervisory prosthesis control. The existing designs of direct EMG control discussed in this section are summarized in table 2 in the appendix along with critical study components (i.e. measured muscle activity, EMG decoding methods, control parameters, level of amputation, and activity used for evaluation).

## 3. Current challenges and opportunities

### 3.1. Questions from motor control perspective and future research directions

The existing literature has shown scattered ideas for using EMG signals to control the robotic lower limb prostheses, from using EMG to switch the prosthesis control mode (supervisory control) to using EMG for continuous control of joint torque (direct control). Despite promising pilot results, the future design of EMG control, in our opinion, should be guided by a systematic framework, built upon theory or mechanistic approaches. We argue that if the goal of EMG control of robotic prostheses is to enable intuitive prosthesis use and bionic function, human motor control theory is a necessary framework to consider.

**Figure 3.**
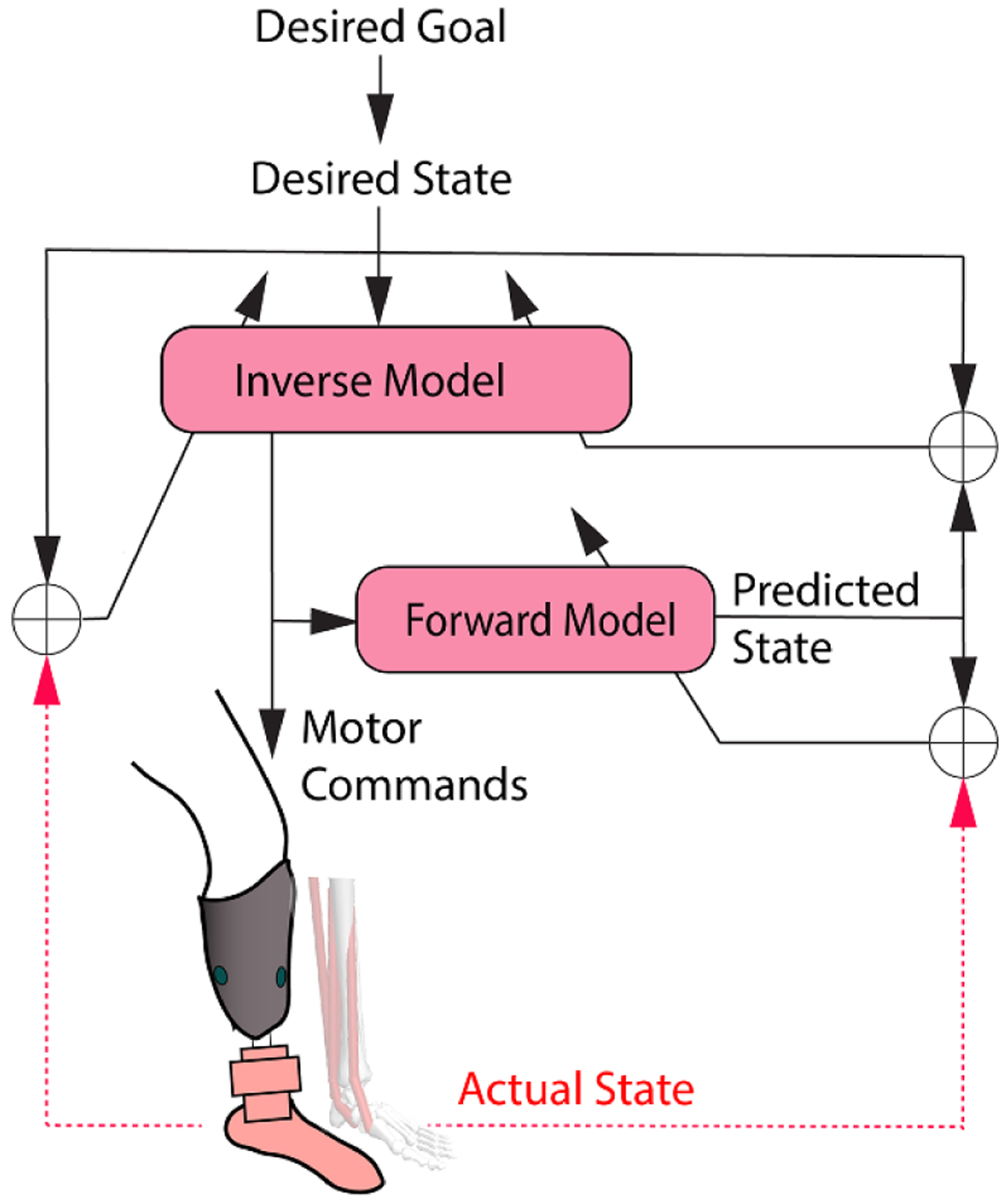
A human motor control framework (adopted from the framework reported in [109] to guide future research in myoelectric control of robotic lower limb prostheses. The actual state of the distal limb is disrupted after limb amputation (red dashed lines). When motor commands (EMG signals of residual muscles) are used to drive a robotic prosthetic limb, humans need to adapt internal model control parameters (the inverse model and forward model) via repetitive motor practice to minimize errors between the desired state and the predicted state, between the desired state and the actual state, and between the predicted state and actual state. This review presents the framework as a means to facilitate future research improving an amputee’s capability to produce appropriate motor commands (residual muscle EMG activity) and control the robotic prosthesis.

Internal models have been one of the established theoretical frameworks to interpret human motor control, although the detailed interpretations of the motor representations and applied computational models vary across groups [109–113]. Here, we adopted the framework reported by Frith *et al* (figure 3), which we use to examine the abnormalities of motor control in different patient populations, including amputees. The key to proficient motor performance is the establishment of a relationship between motor commands and actual system state (i.e. internal models). Note the state hereafter means the human motor control system state as distinguished from the state in a finite-state machine. Awareness of discrepancies among the desired state (related to the goal of the system), actual state, and predicted state enables the update of internal models for improved motor performance via repetitive practice. When biological muscles and skeletons are placed with artificial actuators and machines, the original internal models are disrupted and need to be updated (re-learned) to capture the new relationship between motor commands (including EMG signals of residual muscles) and system state (including state of prosthesis) (figure 3). Hence, we use this framework of human motor control to guide our discussion on open questions and future research opportunities in EMG-based lower limb prosthesis control.

#### 3.1.1. Are lower limb amputees capable of producing needed muscle activity (motor commands in figure 3) sufficiently to learn appropriate internal models for prosthesis control?

In order to apply this motor control framework in figure 3, we must begin by characterizing the possible control inputs that can be used to learn new internal models, post-amputation, for prosthesis control. Because EMG signals are the source of control, answering this question is the key to the success of neural prosthesis control. Many existing studies assume activity of residual muscles is similar to activity of intact muscles. However, this assumption is not necessarily true, evidenced by studies that found abnormalities in residual muscle activation patterns during walking [46, 47]. When asked to voluntarily coordinate the activation of residual antagonistic ankle muscles (i.e. the residual tibialis anterior (TA) and GAS), transtibial amputees showed large variation in their capability to reach certain levels of coactivation [45]. One amputee demonstrated an extensive capability in co-activation of the residual TA and GAS, while some amputees could only activate one muscle at a time. In the latter case, designing a direct EMG prosthesis controller requiring flexible coactivation of antagonistic muscles to function is probably not suitable for these individuals. Similarly, limited co-activation patterns among residual muscles may constrain the number of movement classes distinguishable by an EMG pattern recognition-based decoder.

One fundamental unanswered question is what causes the abnormality and large inter-individual variations in activation and coordination of residual muscles in individuals with limb loss. As previously discussed, surgical techniques and altered peripheral nerve/muscle configurations due to limb amputations can be contributors. In addition, the lack of sensory feedback and discontinued use of the amputated limb causes changes in the motor control system and motor representations, which may modify the feedforward motor commands over time. Evidence providing greater insight into these possibilities has been limited. Further research efforts in understanding the alteration of physiology in residual muscles and the peripheral and central nervous system after limb amputation may unveil the cause of abnormality and variations in coactivation of residual muscles. The results would help find solutions (e.g. a new combination of surgical techniques, implants [114], efferent and afferent neural interfaces) to further improve EMG control (enriched neural information, improved reliability, and voluntary controllability) and determine appropriate and practical EMG decoding designs.

#### 3.1.2. Can lower limb amputees adapt and learn how to produce appropriate EMG activation for prosthesis control (i.e. updates of internal models in figure 3)?

EMG decoding methods might influence the capability of amputees to adapt and reliably use EMG controlled robotic prostheses. Previously, there have been two basic concepts in designing EMG decoders. One uses humans’ adaptability to learn how to use an EMG decoder with a biomimetic and straightforward mapping, such as proportional EMG control [5]. In this case, when errors between desired and actual prosthesis states occur (see figure 3), amputees can learn to adjust the activation level of residual muscles to achieve an intended motion. Another design is based on machine learning algorithms with the hope that artificial intelligence can adapt to the human’s existing EMG activation pattern without requirement of human adaptation, such as EMG pattern recognition [4, 26, 80, 84, 91]. However, such a design might limit human adaptation and learning. For example, in the supervised EMG control, when EMG pattern recognition error happens, users may not even sense the error between desired and actual state of the prosthesis as observed in our previous study [103]. Even if the users can sense the error, how to modify residual muscles’ activity to correct errors is not straightforward to the users because the mapping from residual muscle pattern to movement classes is a black box. Determining appropriate characteristics in EMG decoder design (e.g. continuous vs discrete, black box vs white box mapping) that enable human adaptation will be important for future prosthesis technology. It may even lead to the merging of the two design concepts, leveraging both machine adaptation and human adaptation for faster and more robust EMG prosthesis control.

To our knowledge, in-depth studies examining the systematic training of lower limb amputees in using EMG controlled lower limb prostheses are missing from the literature. These types of studies are needed to understand the potential and limits of EMG control for restoring the motor function of individuals with lower-limb amputations. Collaborations between researchers in physical therapy and engineering are needed to develop effective training methods and protocols that enhance human adaptation in using EMG controlled prostheses for activities of daily living. This line of research could also open many exciting opportunities to address questions related to amputee motor control and learning in gait and posture. For example, exploring adaptations in muscle activation patterns after training in using EMG prosthesis control may reveal neuroplasticity of motor control mechanisms in amputees. Human motor control models [110, 113, 115–117] may explain variations of training effects across individuals and identify potential factors (such as physiological constraints or peripheral nerve injuries) that limit adaptability in using EMG controlled lower limb prostheses. These factors can be also used to predict an individual amputee’s capability for using an EMG controlled prosthesis for improved motor function in the future.

#### 3.1.3. Does EMG control of robotic prosthetic legs increase mental load?

Walking, the most common daily activity involving lower limbs, requires little cognitive effort in able-bodied adults [118]. The question often facing researchers, when designing EMG controlled robotic prosthetic legs, is whether the amputee user has to ‘think’ about how to activate residual muscles and ‘pay attention’ to prosthesis joint motion at all times, which is undesirable because it increases the user’s cognitive load. Additional mental load could be detrimental to postural stability and balance confidence in walking [119, 120], especially for individuals with lower limb amputations since they have already reported the need to ‘concentrate on every step’ [119] without neuromuscular control.

Though cognitive load has been quantified for the lower limb amputee population [121], we are unaware of any research quantifying effects of lower-limb prosthesis control approaches (particularly EMG control approaches) on cognitive load. Supervisory EMG control avoids the need for continuous neuromuscular control [4, 26, 29] and may have advantages in reducing cognitive load. The EMG decoder is discrete and only acts during task mode transitions. Therefore, it does not impose additional mental load to amputees in using neuromuscular control most of the time in walking. For direct EMG control, extra mental processes while learning how to use EMG to operate a prosthesis joint during task performance are initially needed [5, 58, 107]. However, mental load may reduce after training. In addition, as direct EMG prosthesis control mimics human neuromuscular control mechanisms for the biological limb, we postulate that long-term use of direct EMG control may restore original motor pathways for missing limb control and normalize the mental workload needed in lower limb amputees in walking. Testing this hypothesis would enhance our understanding of how training alters human–machine performance in the long run. To achieve this aim, future research needs to identify methodologies and novel technologies (e.g. mobile EEG [122, 123]) that can quantify human cognitive load in locomotion beyond traditional dual task paradigms [124] or questionnaires [125].

#### 3.1.4. Does restoration of haptics and proprioception of prosthetic limbs further assist feedforward neuromuscular control of robotic legs?

This question is motivated by the human motor control framework in figure 3, in which the feedback of actual limb state is necessary but is missing in individuals with lower-limb amputations. In general, the actual lower-limb state is fed back via haptics sensation in the foot and proprioception in lower limbs. Therefore, we discuss these sensory modality individually.

Haptic sensation is important for humans to interact with environments, such as object manipulation via hands [126], identifying terrain type [127], and proper foot placement [128]. For individuals with lower-limb amputations, haptic sensation is reduced with current prosthesis technology. Although haptic sensation of prosthesis foot contact can be received via the residual limb within the socket interface or residual bones via osseointegration [129], the sensation lacks spatial resolution to directly map plantar foot contact areas with the ground. Recent technologies in afferent nerve stimulation [130–132] aim to evoke haptic sensation of the missing feet in individuals with lower limb amputations. A case study has shown promising preliminary results in improving gait stability, energetic efficiency, and cognitive load even with a passive prosthesis [131]. Coupling novel afferent interfaces with feedforward neuromuscular control for closed-loop prosthesis operation has not been fully demonstrated yet. Understanding the effects of haptic sensation of a prosthetic foot on the ability of amputees to coordinate residual muscle activity for EMG prosthesis control is an exciting area for future work.

Proprioception (feedback of joint position, muscle force, and movement) also plays a critical role in human movement control. Artificial afferent nerve stimulations or targeted sensory reinnervation [133] seldomly evoke proprioceptive sensations. The innovative AMI procedure (discussed previously) combined with muscle stimulation has been the most promising method to evoke proprioception so far [48]. A patient case study has shown more normative activity (reduced coactivation and tonic activation) of residual muscles in a transtibial amputee with AMI surgery, partially due to increased proprioception.

For individuals who have no access to the AMI procedure, can they be aware of and predict prosthesis motion? Based on the human motor control framework (figure 3), training of amputees in direct EMG control of a prosthesis itself may aid the sense of prosthesis motion. This is because estimation of the actual motor system state (such as limb movement and position) depends not only on the afferent feedback, but also from the stream of efferent movement commands (efference copy) to the residual muscles (figure 3) [109, 112]. This may explain why amputees can still sense the ‘movement’ the missing limb, even though the peripheral receptors no longer exist. The perception of missing joint movement gradually diminishes due to internal model updates. When the function of efferent motor commands (EMG signals) is restored for prosthetic joint control, through practice, the internal models could be re-built. Therefore, we postulate that through direct EMG prosthesis control and sufficient training of amputees in learning internal models (relationship between EMG commands and action of prosthesis), perhaps amputees could regain awareness and prediction of prosthetic limb movement/position, even without artificial proprioceptive feedback or visual feedback of the prosthetic limb. Additional research is needed to test this hypothesis.

### 3.2. Current challenges from a clinical practice perspective and potential solutions

Since prostheses are assistive devices for daily use, we also want to highlight the research and technology development needed towards making EMG control clinically viable.

#### 3.2.1. Are EMG signals too noisy for daily practice?

Physical disturbances within the prosthetic socket can affect the interface between the skin and the surface EMG electrodes (such as humidity, shift of electrode contact, motions and collision). These disturbances elicit variations in time and frequency components of EMG recordings, interfering with EMG control [134, 135]. One study reported that socket pressure was highly associated with EMG activity of residual muscles [136]. Unfortunately, systematic investigation of the key factors of socket design and fit that affect EMG signals of residual limb muscles is lacking. Maintenance of the EMG interface is critical for successful application of EMG-based prosthesis control in everyday settings. Implantable EMG sensors [61, 63, 137], flexible and stretchable EMG sensors and sensor arrays [69, 138], and new prosthesis attachment methods (e.g. osseointegration [129]), provide potential to eliminate complications caused by physical disturbances of EMG interfaces within a socket and promote EMG-based prosthetic control for daily use. In fact, some exciting feasibility study of implantable EMG sensors for lower limb prosthesis control has already been carried out on a lower limb amputee [64], which shows improve robustness in EMG recordings for real-time prosthesis control compared to surface EMG recordings.

EMG signals are random processes often deemed noisy and unreliable for prosthesis control. We argue that the reliability of EMG decoders depends on what and how information in the EMG signals is extracted. As reviewed, information of EMG signals used for prosthesis control has been extracted by various features (e.g. mean absolute value, number of zero crossings, median frequency of the power spectrum density), estimated from the signals in a time window. Some features are sensitive to the aforementioned physical or physiological disturbances, while other features can be more resilient to these disturbances [99]. Identifying reliable EMG features more responsive to user movement intent and less sensitive to noise and disturbances can further improve the robustness of EMG decoders. One promising feature is the firing rate of motor units captured by high-density surface EMG recordings [139, 140]. This feature counts the number of motor unit action potentials per time bin, which is less influenced by signal noises or magnitude and frequency drift. It is a promising method to address the reliability of EMG decoding methods. It is not hard to imagine that a soft, high-density EMG grid could be built into the prosthesis liner for prosthesis control in the future. Another potential method is deep learning, which can automatically learn features for accurate and reliable classification. This method has been explored in EMG pattern recognition for upper limb motion classification recently [141], and can be extended into EMG signals in lower limbs. In addition, various random signal processing techniques and fault-tolerance mechanisms [134] can be explored in the future to address the robustness of EMG control of robotic prostheses.

#### 3.2.2. Are EMG controlled prostheses safe to use?

The failure of lower-limb prosthesis control might lead to falls and injuries in lower-limb amputees. Understanding the amputee user’s safety when relying on an EMG controlled prosthesis is essential to evaluate the device’s practical value. Previous studies have investigated effects of EMG pattern recognition errors selecting locomotion mode [101, 103] and identified a set of critical pattern recognition decision errors that disturb the user’s walking stability and perceived stability. Future work should focus on how to eliminate these critical errors to ensure the user’s balance and safety. For continuous, direct EMG control, the error correction and tolerance become the responsibility of the human motor control system [142]. Future research may focus on how to train individuals with lower-limb amputations to calibrate the forward model (figure 3) for error tolerance and correction when performing tasks with direct EMG control of a robotic prosthetic leg.

#### 3.2.3. What are the benefits and limitations of EMG prosthesis control, compared to existing autonomous prosthesis control, for daily prosthesis use?

Understanding the benefits and limitations of EMG prosthesis control, compared to the existing autonomous approach, is necessary for future clinical translation. In terms of function, robotic machines are good at tasks in known contexts with precision and fast feedback control rates but lack adaptability and flexibility. On the other hand, human motor control systems are slow and have large variation in movement output but are highly adaptable to deal with varied environments and versatile activities. This view is applicable to autonomous control versus EMG-based control of robotic lower limb prostheses. Existing autonomous controllers are very reliable for biomechanically well-established, stereotypical tasks (such as walking). However, they are inadequate to handle unstructured daily environments and activities that cannot be easily predicted or pre-programmed. On the other hand, EMG control of lower-limb prostheses enables versatile prosthesis function adaptive to various contexts, but control can be limited by the lack of accuracy and capability of amputees to produce needed EMG control signals. One way to explore the benefits and limits of the two approaches in the future is via task allocation [143]. We can classify whether a task can be achieved by only one method or both autonomous and human neuromuscular control; in the latter case, additional research should compare the two methods on system design complexity and the user’s task performance. The gained knowledge could guide future design of robotic lower-limb prosthesis control, shared by both autonomous and human motor controllers.

In terms of utility and user acceptance, we should also consider evaluating both autonomous and EMG control methods by measuring the user’s learning rate, cognitive workload, trust in the robotic prosthesis, satisfaction, and sense of embodiment. To our knowledge, these user-centered evaluations have not been systematically quantified and reported, even on existing autonomous prosthesis control schemes. Collaboration with researchers in cognitive ergonomics and clinical outcome measurements are needed to evaluate the user’s acceptance and utility of various controllers for robotic lower limb prostheses.

While the challenges and opportunities we have discussed here are important, this is not an exhaustive list. Several factors, such as device cost, limited prosthesis reimbursement, and power requirements stand as challenges in the path translating new prosthesis technology to end users. The introduction of EMG control will likely face these challenges as well. However, the benefits of EMG control to prosthesis function, reviewed here, demonstrates the value of continued investment in its development. Further, these challenges likely do not all need to be addressed before this control can begin to be introduced in new lower-limb prosthetic technology.

## Data Availability

No new data were created or analyzed in this study.

## Appendix

**Table 1.** Supervisory EMG control review summary.

Author^a^	Muscles recorded^b^	Classifier type^c^	EMG features	Windowing/prediction time	Training	Control type	Amputation type	Activity
Au *et al* [81]	TA, GAS, SOL	Neural network	Pre-processed EMG	Predictions at 0.5 Hz	Back propagation method with pre-processed EMG as input	None; offline classification only	Transtibial	Trajectory tracking sitting
Jin *et al* [76]	ADDL, TFL, RF, VL, VM, BF, SM, ST	Distribution of different muscle features	Mean absolute value, waveform length, mean square value, zero tangent number, median power frequency	Entire gait cycle	3–5 times along walkway for each condition	None	Transfemoral	Level-ground walking at varying speeds, stairs, and ramps
Huang *et al* [4]	GME , GMA , SAR, RF, VL, VM, GRA, BFL, BFS, SEM, ADM	LDA and neural network	3rd order autoregression coefficients, mean absolute value, zero crossings, waveform length, number of slope sign changes and root mean square	Range from 50 to 150 ms EMG window, 200 ms phase window	Ten walking trials of all activities with own prosthesis	None; offline classification only	Transfemoral	Level-ground walking, stepping over and obstacle, stairs, ipsilateral and contralateral turning, and standing
Au *et al* [11]	GASL, GASM, TA	Neural network	Variance of EMG signals	100 ms EMG window	Matching imagined ankle position to virtual ankle	Impedance control	Transtibial (bilateral)	Level-ground walking, stair descents
Ha *et al* [94]	Quadriceps, hamstring	LDA, QDA	Not provided	Not provided	100 s of knee flexion/extension visualizations	Online impedance control (prosthesis next to participant)	Transfemoral	Virtual tracking tasks (sitting)
Huang *et al* [26]	GME, GMA, SAR, RF, VL, VM, ST, GRA, BFL, BFS, ADM	SVM, LDA	Zero crossings, signal direction change, mean absolute value, waveform length	150 ms sliding EMG window prediction	15 times each activity	None; online classification	Transfemoral	Walking, stair/ramp ascent and descent, stepping over obstacles (and transitions)
Simon *et al* [144]	SM, SAR, TFL, ADM, GRA, RF, VL, BFL	Two LDA classifiers	Mean absolute value, zero crossings, waveform length, slope sign change number, five-count majority vote	250 ms overlapping	Ten sit-to-stand trials	Impedance control	Transfemoral	Sitting down, walking and transition between them, repositioning prosthesis while sitting
Hargrove *et al* [29]	Two reinnervated muscle segments, proximal BF, RF, VL, VM, SAR, GRA, ADM, TFL	DBN	Not provided	Not provided	Offline, 20 reps of locomotor circuit	Impedance control	Transfemoral	Level-ground walking, ramps, stairs and outside stairs
Young *et al* [145]	ST, BF, TFL, VM, SAR, ADM, GRA	Two LDA models	Mean absolute value, waveform length, zero crossings, slope sign change, 1st two coefficients of 3rd order autoregressive model	300 ms before heel contact and toe off	Offline, 20 reps of two locomotor circuits, train classifier on three subjects test 1 as novel user. Then add 5–10 min of level-ground walking for novel user to train classifier.	None; offline classification	Transfemoral	Level-ground walking, ramps, stairs
Zhang *et al* [78]	RF, VL, VM, TFL, BFL, BFS, ST, ADM	SVM	Zero crossings, slope change number, mean absolute value, waveform length	EMG window 150 ms decision time between 45 and 28 ms	Offline training, at least five reps of 30 s of data per condition	None; online classification	Transfemoral	Ramp/stair ascent, descent, level-ground walking, sitting, standing
Tkach *et al* [91]	TA, PL, GASL, GASM	LDA	Mean absolute value, zero crossings, slope sign change, and waveform length	250 ms window	12 trials of each ambulation mode (stairs and ramps), and 12 trials of level-ground walking	None; offline classification only	Transtibial	Level-ground walking, stairs, ramps, and transitions
Tkach *et al* [84]	TA, PL, GASL, GASM, VM, VL, RF, BF	LDA	Six coefficients from 6th order autoregressive model, mean absolute value, zero crossings, slope sign change, waveform length	250 ms prediction window 50 ms overlap	18 s for data for each movement class (one-DOF up to three-DOF, including no movement flexion, rotation, in/eversion)	None; Offline classification only	Transtibial	Virtual tasks for varying DOF ankle movements (rotation, flexion, in/eversion)
Hargrove *et al* [146]	ST, SAR, TFL, ADM, GRA, VM, RF, VL, BFL	LDA	Mean absolute value, zero crossings, slope sign change, waveform length	250 ms window, decisions every 50 ms	EMG for virtual cases, four reps of 3 s for each movement, no feedback provided	Online impedance control and virtual avatar feedback	Transfemoral	Knee flexion/extension, ankle plantar flexion/dorsiflexion,internal/external tibial/femoral rotation, relaxation
Miller *et al* [80]	TA, GASM, VL, BF	LDA and SVM	Mean absolute value, variance, wavelength, number slope sign changes, zero crossings	Three subwindows with varying times based on heel strike and toe off	Six trials each activity, additional trials for different walking speeds	None; offline classification only	Transtibial	Level-ground walking three speeds, ramp/stair ascent/descent
Du *et al* [97]	SAR, RF, VL, VM, GRA, BFL, ST, BFS,	Adaptive algorithm: EBA and learning from test data	Absolute value, slope sign changes, waveform length, zero crossings	160ms sliding window	15–10 trials of each activity	None; offline classification only	Transfemoral	Level-ground walking, ramps, stairs and their transitions
Zhang *et al* [147]	RF, VL, VM, BFL, SAR, ST, ADM	Not provided	Not provided	Not provided	Offline training with ten reps of walking level-ground and ramp course	Impedance control	Transfemoral	Standing, level-ground walking ramp ascent/descent
Young *et al* [148]	ST, BF, TFL, RF, VL, VM, SAR, ADM, GRA	DBN	Mean absolute value, waveform length, zero crossings, slope sign change, two autoregressive coefficients	Tested 0–450 ms windows with 20 ms sliding window	Offline training with 20 reps of locomotor circuit	None; offline classification	Transfemoral	Level-ground walking, ramps, stairs
Hargrove *et al* [85]	ST, BF, TFL, RF, VL, VM, SAR, ADM, GRA	LDA + DBN	Not provided	Not provided	Offline training, 20 reps of locomotor circuit, level-ground walking at variable speeds, stopping/starting/turning	Impedance control	Transfemoral	Walking, stairs, ramps
Spanias *et al* [149]	ST, ADM, TFL, RF	DBN	Mean absolute value, waveform length, zero crossings, slope sign changes, six autoregressive coefficients	300 ms window	20 reps of locomotion circuit	None; offline classification only	Transfemoral	Level-ground walking, ramp/straight ascent/descent, turn around
Spanias *et al* [102]	ST, BF, TFL, RF, VL, VM, SAR, ADM, GRA	LDA with log-likelihood	Mean absolute value, waveform length, zero crossings, slope sign change, 1st two coefficients of 3rd order autoregressive model	300 ms before toe off and heel strike	Offline training with 20 reps of locomotor circuit	Impedance control	Transfemoral	Level-ground walking, ramps, stairs
Liu *et al* [100]	RF, VL, VM, BFL, SM, TFL, BFS	EBA, TSVM	Absolute value, signal length, slope sign changes, zero crossings	160 ms window avg processing time 45 ms	Four 1 min trials of each locomotion mode collected in separate training session	EBA online impedance control	Transfemoral	Level-ground walking, ramps, stairs, and their transitions
Spanias *et al* [92]	Pairs of electrodes over RF, TFL, ST, ADM	Eight DBN classifiers + LDA	Mean absolute value, waveform length, zero crossings, slope sign changes, six autoregressive coefficients from 6th order model PCA and ULDA to features totals	Transition modes 300 ms (210 ms before gait event and 90 ms after gait event)	Offline training with 1st session data across locomotor modes. Online training 2nd session through forward prediction.	Impedance control	Transfemoral	Level-ground walking, ramps, stairs over multiple days
Hussain *et al* [77]	GM, GASL, GASM, TFL, RF, VL, VM, BFL, SOL, TA	SVM and LDA	Bispectrum for high order frequency spectrum/non-Gaussian info, RMS, zero crossings, histogram, integrated EMG, sum of square integral, waveform length, mean/median frequency, autoregression, and reduced with PCA	Non-overlapping 150 ms-300 ms windows	Offline training five trials for each walking mode	None; offline classification only	Transtibial and transfemoral	Slow, steady state, and fast walking, ramps

a Authors are listed in order of year published.

b Muscle abbreviations: adductor magnus (ADM), adductor longus (ADDL), biceps femoris (BF), biceps femoris long head (BFL), biceps femoris short head (BFS), extensor digitorum brevis (EDB), gastrocnemius (GAS), gastrocnemius medialis (GASM), gastrocnemius lateralis (GASL), gluteus maximus (GMA), gluteus medius (GME), gracilis (GRA), peroneus longus (PL), rectus femoris (RF), sartorius (SAR), semimembranosus (SM), semitendinosus (ST), soleus (SOL), tensor fasciae latae (TFL), tibialis anterior (TA), vastus lateralis (VL), vastus medialis (VM).

c Classifier abbreviations: support vector machine (SVM), neural network (NN), linear discriminant analysis (LDA), quadratic descriminant analysis (QDA), dynamic Bayesian network (DBN), entropy-based adaption (EBA), transductive support vector machine (TSVM).

**Table 2.** Direct EMG control review summary.

Author^a^	Muscle^b^	EMG decoding method	Modulated control parameters	Amputation type	Activity
Ha *et al* [94]	Quadriceps, hamstring (unspecified)	Envelope (2 Hz cutoff), 20% MVC threshold QDA	Reference angular velocity	Transfemoral	Virtual tracking task (sitting)
Hoover *et al* [107]	VL, VM, RF, BF	Envelope (5–10 Hz cutoff).	Flexion/extension torque	Transfemoral	Level-ground walking
Hoover *et al* [104]	BF, RF, ST, VL, VM	Envelope (2.5 Hz cutoff)	Flexion/extension torque	Transfemoral	Stair ascent
Dawley *et al* [27]	Quadriceps, hamstring, (unspecified)	Initial processing not described, principal component analysis (flexion/extension)	Reference angular velocity, joint stiffness	Transfemoral	Level-ground walking
Wang *et al* [28]	GAS (unspecified head)	Low-pass filtered (15 Hz), rectified envelope (200 ms moving average window)	Plantar flexor torque gain (push-off only)	Transtibial	Level-ground walking
Alcaide-Aguirre *et al* [150]	TA	Envelope (10 Hz cutoff)	Virtual object acceleration	Transtibial	Virtual tracking task (sitting)
Chen *et al* [151]	TA, GAS (unspecified head)	Envelope (2.5 Hz cutoff), PCA (flexion/extension)	Reference angular velocity, joint stiffness	Transtibial	Virtual target hitting (sitting)
Huang *et al* [105]	GASL	Envelope (2 Hz cutoff)	Pneumatic artificial muscle force	Transtibial	Level-ground walking
Huang *et al* [5]	GASM or GASL	Envelope (2 Hz cutoff)	Pneumatic artificial muscle force	Transtibial	Level-ground walking
Huang *et al* [152]	TA, GASM or GASL	Envelope (2 Hz cutoff)	Virtual object position	Transtibial	Virtual ballistic target hitting (sitting)
Clites *et al* [48]	TA, GASL, TP, PL	Envelope (100 ms moving average window)	Flexion/extension torque	Transtibial	Virtual target hitting, stair ascent/descent, obstacle walking
Fleming *et al* [23]	TA, GASL	Envelope (2 Hz cutoff)	Virtual spring stiffness	Transtibial	Virtual balancing inverted pendulum (sitting)
Huang *et al* [45]	TA, GASL	Envelope (2 Hz cutoff)	Virtual cursor position	Transtibial	Virtual control input space filling (sitting)
Dimitrov *et al* [71]	TA, GASM, GASL	Envelope (5 Hz cutoff), non-negative matrix factorization (125 ms windows)	Equilibrium angle, joint stiffness	Transtibial	Target hitting (standing), walking (with passive device)
Fleming *et al* [58]	TA, GASL	Envelope (2 Hz cutoff)	Pneumatic artificial muscle force	Transtibial	Quiet standing (vision, no vision, foam and firm surfaces), Sit-to-stand, load transfer.

a Authors are listed in order of year published.

b Muscle abbreviations: biceps femoris (BF), gastrocnemius (GAS), gastrocnemius medialis (GASM), gastrocnemius lateralis (GASL), peroneus longus (PL), rectus femoris (RF), semitendinosus (ST), soleus (SOL), tibialis anterior (TA), vastus lateralis (VL), vastus medialis (VM), tibialis posterior (TP).
